# Green Minimalistic Approach to Synthesize Chitosan-Based Durable Polymer Hydrogel Materials for Supporting Cell Growth

**DOI:** 10.3390/gels11070485

**Published:** 2025-06-23

**Authors:** Justyna Pawlik, Klaudia Borawska, Piotr Wieczorek, Kamil Kamiński

**Affiliations:** 1 Department of Glass Technology and Amorphous Coatings, Faculty of Materials Science and Ceramics, AGH University of Science and Technology, 30 Mickiewicza Ave., 30-059 Krakow, Poland; pawlikj@agh.edu.pl; 2 Faculty of Chemistry, Jagiellonian University, Gronostajowa 2 St., 30-387 Krakow, Polandpiotr.wieczorek@doctoral.uj.edu.pl (P.W.); 3 Doctoral School of Exact and Natural Sciences, Jagiellonian University, Lojasiewicza 11, 30-348 Krakow, Poland

**Keywords:** chitosan, cell support, biodegradable plastics

## Abstract

In this work, we present an innovative, crosslinker-free method for preparing chitosan-based hydrogel precursors, fully aligned with green chemistry principles and composed of only five non-toxic, readily available reagents. The key novelty lies in the use of glycerin, which, during thermal annealing, evaporates and triggers a surface or bulk chemical transformation of chitosan, depending on its concentration. This process significantly enhances the material’s mechanical properties after swelling—with up to a 35% increase in tensile strength and a notable reduction in water uptake compared to systems containing AMPS-based crosslinkers. FTIR analysis indicates a partial re-acetylation of chitosan, shifting its structure toward that of chitin, which correlates with improved hydrophobicity (as shown by increased contact angles up to 92°) and greater structural integrity. These improvements are particularly pronounced at glycerin concentrations of 10–20%, whereas higher concentrations (50%) result in brittle, non-moldable films. Importantly, preliminary biological tests confirm that the resulting hydrogels are effectively colonized by mammalian cells, making them promising candidates for bioimplant or tissue engineering applications. Surface morphology and compatibility were further assessed via SEM, AFM, and contact angle measurements.

## 1. Introduction

Non-biodegradable plastics derived from fossil fuels represent some of the most difficult waste to manage [1]. Their lifespan in nature without human intervention is hundreds, if not thousands, of years [2], while all management tactics seem to be environmentally unsafe (incineration/burning) or often economically unfeasible (recycling) [3]. These problems, combined with the growing threat of increasing the presence of microplastics in drinking water [4] (resulting from the mechanical degradation of such polymers), result in a widespread trend aimed at replacing the currently used utility and biomedical polymers with natural and biodegradable ones [5].

In the case of polymers used in biomaterials, the condition of biodegradability is clear, while the origin of the raw material is not as obvious [6]. The majority of polymeric materials used in medicine today have been derived in one way or another from fossil fuels, and this constitutes their original sin from the perspective of green and sustainable chemistry. This is mainly due to the ease of processing and resistance to physical and chemical sterilization, which is not so good with natural polymers [7]. Specifically, for materials that are polymeric hydrogels consisting mainly of water, susceptibility to evaporation at high temperatures or contamination during chemical sterilizing agents (e.g., ethanol) [8] is a problem. Therefore, our proposed materials show resistance to ethanol and temperature, which are used during synthesis, and at the same time, ensure the sterility of the obtained material from the start.

When it comes to cell culture scaffolds that would ultimately serve as implants after colonization by cells, a critically important parameter that describes them is mechanical strength [9]. We are not only talking about mechanical strength in dry form but also after swelling with an aqueous solution or a dedicated medium [10]. Due to the fact that cultures are conducted in a cell medium and that there is a need to supply cells with water, materials of this type will be used in practice in this form. Good mechanical durability in this form is necessary so that they can be attached to the regenerated tissue, whether by sutures or surgical adhesives [11].

Hydrogels are a natural candidate for cell growth substrates and 3D scaffolds [12]. Due to the significant proportion of water in their mass, they reproduce a natural share of this liquid in tissue [13] and substrates based on plant-derived polysaccharide hydrogels, such as agarose, which have been used as substrates in microbiology for decades [14]. In addition to the biopolymers naturally associated with hydrogels, such as agarose, gelatin, or starch, chitosan seems to be an interesting alternative for developing materials for such applications.

Chitosan is a deacetylated derivative of chitin [15]—a polysaccharide that builds the exoskeletons of insects and crustaceans [16]. This makes the substrate widely available, while the reagents used in the deacetylation reaction (NaOH solution) [16] are simple and, if used properly, non-toxic to the environment. Chitosan has been widely used in the research of new biomaterials, and its great potential in these applications is postulated [17,18]. As a non-protein macromolecule, it has negligible immunogenicity but has NH_2_ [19] groups in its structure (characteristic of proteins) that are desirable in many applications. It has limited solubility at a physiological pH, while it dissolves well in acidic environments, giving viscous solutions that can be effectively converted into hydrogels by crosslinking [20]. Optimal crosslinking compounds for chitosan have been the subject of research for several decades, starting with the first used but toxic glutaraldehyde [21], biocompatible plant-derived genipine [22], and other recently developed systems. In the case of chitosan, the issue of selecting a crosslinking agent can be considered a problem, and therefore, one of the goals of this work is to try to propose a minimalist system that does not require this element.

As we have shown, it is possible to obtain a hydrogel with good mechanical properties from chitosan without chemical crosslinking, implementing the principles of green sustainable chemistry [23]. The minimal number of reagents that can be obtained from natural sources (glycerin, acetic acid, ethanol, and sodium carbonate) used routinely in applications ranging from food to cosmetics makes the proposed procedure promising from the perspective of practical applications. For many new green technologies, it is the degree of complexity that is the obstruction because, combined with the cost, it represents an impossible constraint to adapt to larger-scale productions [24]. Most importantly, the materials obtained using our innovative procedure have good, acceptable mechanical properties (similar to those of widely used polymers such as PP and PE) and, in terms of interactions with water (relatively high contact angle, low swelling), promote cell growth.

## 2. Results and Discussion

### 2.1. Macroscopic Form of Materials

In this paper, to demonstrate the superiority of the new method for obtaining an innovative hydrogel material, we compared three strategies for converting a chitosan solution into a polymer hydrogel precursor—a material that, after swelling, becomes a ready-to-use substrate. All of these tactics assumed the use of a minimal amount of additional substances necessary to convert crude chitosan into a finished material. The first reference synthesis strategy involved the introduction of an acid derivative of methacrylic acid (AMPS) that simultaneously acts as an acid to improve the solubility of chitosan and as a crosslinking agent. This system, after the initial drying phase and the introduction of a small amount of radical polymerization initiator, was transformed at 70 °C into a relatively stable polymer hydrogel (LMWChAMPS). Such crosslinking by radical polymerization is a standard technique used to improve the properties of hydrogels, in particular those containing amine groups (e.g., in PAGE hydrogels). The second tactic involved neutralizing the semi-dried material with a solution of K_2_CO_3_ in ethanol and then annealing the material at 170 °C (LMWCh or MMWCh). The prefixes LMWCh and MMWCh are derived from low-molecular-weight and medium-molecular-weight chitosan. The third strategy was an expansion of the second one by adding glycerin in various amounts (10, 20, or 50%) to the system at the drying stage (LMWChG10, LMWChG20, or LMWCh50). This allowed us to explore an additional objective of the work, namely, to study the effect of this additive on the finished material. It should be emphasized here that neutralization and annealing of the material are necessary operations for obtaining a stable material if a crosslinking agent is not used, as indicated in our earlier studies. The positive effect of glycerin on the mechanical properties of chitosan-based materials has already been reported in the literature, but there are no reports of combining glycerin with sample heating or using glycerin without a crosslinking agent.

**Figure 1:**
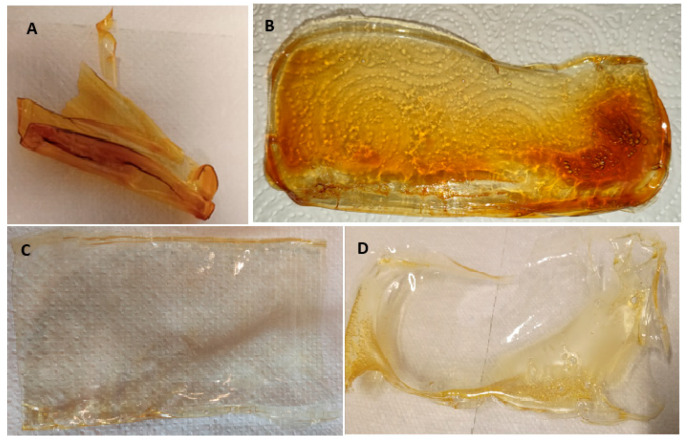
Macroscopic images of the selected materials obtained. (**A**) LMWChG50; (**B**) LMWChG20; (**C**) LMWCh; (**D**) LMWChAMPS.

The figure below (Figure 1) includes pictures taken without magnification of the ready materials. They take the form of thin films about 0.5 mm thick and with a yellowish tint (AMPS) transitioning to brown, as seen below (chitosan only).

During drying, most of the obtained materials were not significantly degraded or wrinkled and were macroscopically smooth (Figure 1B–D). The exemption is one containing the highest amount of glycerin (LMWChG50) (Figure 1A). This material undergoes multiple fragmentation due to the stresses that occur during drying, and the individual fragments are twisted and deformed. This means that, for this system, it is impossible to carry out some of the further experiments for this sample.

### 2.2. Surface Microimaging Using SEM Microscopy

**Figure 2:**
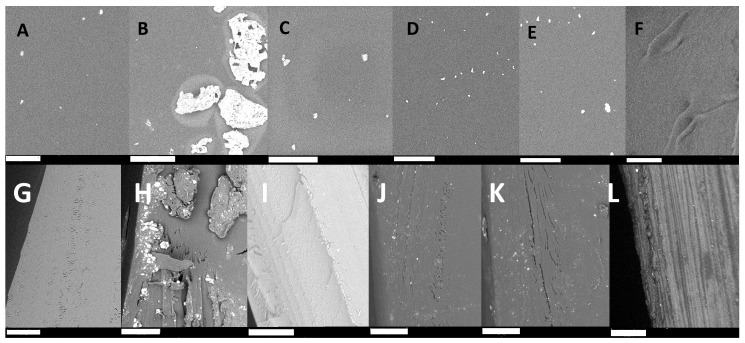
Surface images of all materials described in this paper taken using SEM microscopy (**A**) LMWCh, (**B**) MMWCh, (**C**) LMWChAMPS, (**D**) LMWChG10, (**E**) LMWChG20, and (**F**) LMWChG50, and cross-sections (**G**) LMWCh, (**H**) MMWCh, (**I**) LMWChAMPS, (**J**) LMWChG10, (**K**) LMWChG20, and (**L**) LMWChG50. Note: 30 micrometer size bar.

Macroscopic characterization of the obtained materials described in the previous section was supplemented by imaging their surfaces using SEM microscopy (Phenom World RO PIK INSTRUMENTS, Piaseczno, Poland) (surfaces and cross-sections). The use of an environmental scanning electron microscope and the fact that the material is in the form of thin films allowed us to image the samples without additional processing, which gives an image that fully reflects the true state of the surface. Below are figures (Figure 2) showing representative images of surfaces and cross-sections of all the materials obtained.

The images presented above (Figure 2) show that the surfaces of the materials obtained are very similar for the samples LMWCh, LMWChAMPS, LMWChG10, and LMWChG20 (Figure 2A,C–E) and are flat with relatively high homogeneity except for a small number of rare objects of about 1 µm in size. These objects are probably the remains of insoluble impurities or not completely reacted chitin presnt in natural raw material. Two materials with distinguishable surface characteristics stand out in the compilation above. The first is the MMWCh material (Figure 2B), which contains analogous non-homogeneous objects, as in the samples described earlier, but that are much larger and occur more frequently, covering almost the entire surface. This can be explained by the use of a different chitosan, which not only differs in mass but may also differ in the amount of impurities. Alternatively, this difference can be explained by the inferior solubility of chitosan with a higher mass, and the visible objects are simply lumps of undissolved chitosan. It could also be the sum of these two effects, but it is undeniable that this material is the most non-homogeneous, which has its manifestations in subsequent results showing its lower mechanical strength. The last surface not matching the others is LMWChG50 (Figure 2F), for which one would expect analogies with other materials derived from low-molecular-weight chitosan; however, this is not the case. In the case of this sample (LMWCh50) (Figure 2F), we do not see any additional objects, and the surface is homogeneous; however, it is not flat, and ripples can be seen. These can be identified with microscopic manifestations of phenomena that cause the material to fold strongly on the macroscopic scale after drying (Figure 1A). This effect is certainly related to the presence of glycerin during the synthesis of the material and is related to its quantity because it occurs only in the system where it is most abundant. It can be identified directly with the reactions that occur on the surface of the material (described in more detail in Section 2.7, and it is in the case of this sample, due to the largest amount of glycerin, it occurs in the most intense way.

In the case of cross-sections, the images obtained can be grouped in almost the same way as views of the surfaces of the obtained materials. Cross-sections of LMW chitosan-based materials—with the exception of those obtained with the addition of the monomer (LMWChAMPS) and that contain the highest amount of glycerin (LMWChG50)—look very similar and are relatively homogeneous, with minor, small, free space/cracks inside the material. The sample with the highest amount of glycerol added during synthesis has a layered cross-section and, as in the case of surface images, stands out from the rest of the materials. This may suggest a non-uniform chemical composition, confirming the occurrence of chitosan acetylation. The material containing AMPS is the most homogeneous, which may be due to the absence of annealing in the manufacturing process. The significant image’s contrast results from the highest electrical conductivity of this material (PAMPS exhibits high proton conductivity). As in the case of surfaces, the MMWCh material also contains large inhomogeneous objects in the cross-section, which again explains its poorer mechanical properties.

### 2.3. Study of the Effect of Water on the Material—Swelling and Contact Angle Measurements

**Figure 3:**
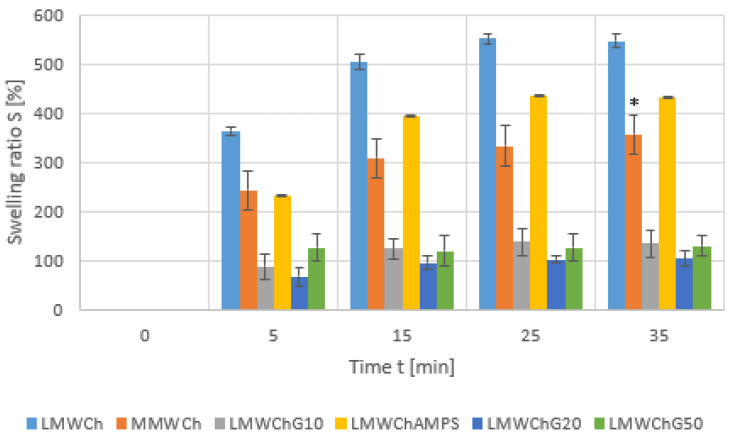
Results of changes in swelling coefficients for the tested materials as a function of time. *—partial disintegration of the material.

Swelling is one of the most important properties of biomaterials based on polymer hydrogels. Swelling is often accompanied by a change in the geometry of the material relative to the initial structure and a deterioration in mechanical properties. For most materials of this type, this is a desirable process so that the retention of water necessary for living components is possible. However, the amount of water solution that is absorbed and the time of this process cannot be too high. It is known that extensive swelling can lead to brittleness or even dissolution of the hydrogel material. Therefore, measurements to quantify these phenomena were the next experiments to which the materials proposed in this paper were subjected, and the results obtained are shown in the graph below (Figure 3).

The figure above (Figure 3) presents the progress of swelling in time for all materials described in this publication. In general, the obtained chitosan materials show moderate swelling. Since these materials are dehydrated at the stage of synthesis, there are, realistically, polymer hydrogels in potency, and the process of swelling is needed so that they can constitute a scaffold for cellular growth. However, it must be a relatively rapid process and of restricted scale to limit the disruption of the geometry and potential disintegration. The MMWCh swells more poorly than LMWCh, but this swelling is accompanied by degradation/disintegration at the 35 min time point. The introduction of an AMPS monomer also partially reduces the swelling, but the smallest swelling occurs in the materials obtained using glycerine. Regardless of the amount of glycerin added, the weight gain during swelling ranks around the 100% value, i.e., twice the initial weight, which distinguishes these materials.

In addition to swelling, the contact angle is the second important parameter, indicating the material’s interaction with water and aqueous solutions. Therefore, this measurement was made for the obtained materials, which are flat films for which such a measurement is technically possible. Contact angle measurements for LMWChG50, due to the geometry of the material, which made this impossible to perform, were omitted. In the case of the MMWCh with a strongly non-homogeneous surface (Figure 2B) that partially degrades during swelling, this measurement was also not performed. For the remaining materials, measurements were made for dry systems and after swelling in water for 35 min, which corresponds to full swelling. The results obtained, which are the average of at least 10 repetitions, are summarized in the table below (Table 1).

An analysis of the results in the table above shows that the distinguishing feature of the materials obtained using glycerin is the persistence of a relatively large contact angle when comparing dry and swollen materials. The fully reliable results in this set that should be considered are the ones obtained for a swollen material because, in the case of a dry material during measurement, the swelling present can distort it (hence, such a large but unlikely physical value of the LMWCh material). Values of around 70 degrees, obtained for the swollen LMWChG materials, indicate a surface that is much more favorable to cell growth than those close to zero for chitosan alone (see the results in Table 1, supplemented by the measurement of a plastic dish fragment dedicated to cell and tissue culture).

### 2.4. Material Strength Tests

The results describing the mechanical strength of the obtained materials are summarized in Table 2. The based materials, i.e., LMWCh and MMWCh, presented satisfactory mechanical properties that correspond to the literature reports [25] with a tensile strength of 20–60 MPa and an elongation at break of 5–15%. However, Young’s modulus (E) values of LMWCh (2870.9 ± 544.4 MPa) and MMWCH (3166.5 ± 471.7 MPa) are significantly improved in comparison with the literature data [A], i.e., E 500–1500 MPa. This proves the higher stiffness of the prepared materials. When it comes to the chitosan material (LMWCh) augmented with glycerin, the values of Young’s modulus decreased, but no significant influence on this parameter (E) was found when comparing the 10 or 20% glycerin modification. Clearly, the addition of glycerine resulted in a less stiff structure with good mechanical properties maintained. The highest values of elongation at break were observed with a 20% glycerin modification.

The visible impact of modification of LMWCh on the tensile properties was observed in the presence of the AMPS monomer. Probably, in this case, the structure was even more flexible, according to the decrease in E and tensile strength (σM) values with relatively high elongation at break (εM) values and maximum force (Fm) values. It is well known that in hydrogels, the hydration state greatly affects mechanical properties. In the case of the LMWCh swollen samples, we observed a rapid decrease in mechanical properties when compared to those observed in the as-prepared LMWCh sample. However, these values still correspond with the literature reports [26,27]. Interestingly, the Young’s modulus of the swollen LMWChG10 was almost twice as high as that of the LMWCh swollen material. It seemed that the addition of glycerine affected the resistance to water absorption by the material.

### 2.5. AFM

To complete the surface characteristics for the selected materials (selected for cell studies), the materials were imaged in dry and swollen form using atomic force microscopy (AFM). For these experiments, we selected the material that was ultimately used as a scaffold for cell growth (LMWCh10) in the next stage of the work, as well as a reference material obtained in an analogous manner but without the addition of glycerin (LMWCh).

**Figure 4:**
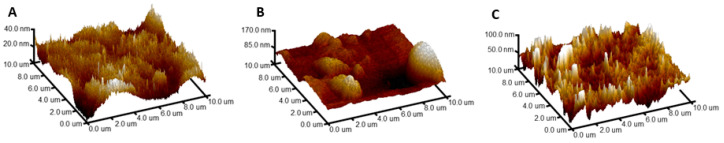
AFM 3D topographic (10 × 10 µm) images of LMWCh (**A**), LMWChG10 (**B**), and LMWCh10 swollen (**C**).

There is a significant difference in surface properties between LMWCh and LMWChG10 (the image of the surface in Figure 4, and the quantitative parameters describing it in Table 3 and Figure S1), indicating that the presence of glycerin during drying affects these properties dramatically. Between the materials, there is an approximately 5-fold increase in roughness, as well as an increase in adhesion and a decrease in elastic modulus (the material becomes softer). At the same time, it was noted that when the material is modified in the presence of glycerin, a decrease in the contact angle is observed (Table 1). According to Wenzel’s model, this suggests that glycerol modification introduced polar functional groups, thus increasing the hydrophilicity of the surface. After subsequent swelling of the LMWChG10 material, the surface was smoothed, adhesion increased, and the modulus of elasticity decreased further. According to this model, after smoothing the hydrophilic surface, the contact angle should increase, which was also observed (Table 1).

### 2.6. Studies Using Cell Lines

**Figure 5:**
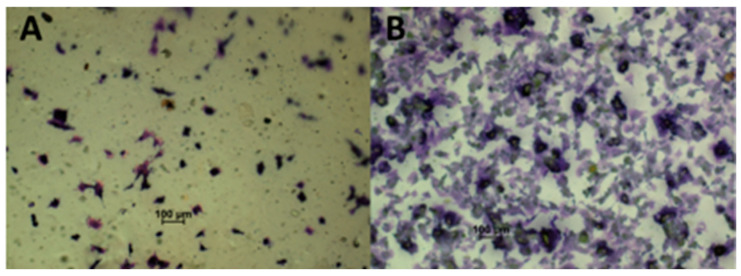
Image of cells stained with crystal violet after 24 h culture on the material: (**A**) LMWCh and (**B**) LMWChG10.

The differences in the physicochemical properties of the materials obtained in the presence of glycerin manifested not only in changes in the interaction with water and in the form of differences in the AFM images of the surface but also in the interaction with the biological system. The images below show the differences between the number of cells on the chitosan materials obtained in the presence of glycerol (10%) (Figure 5B) and in its absence (Figure 5A). This material was chosen because it had the best properties in terms of mechanical properties and contact angle, and at the same time, the number of reagents used was the lowest, which is in line with the assumptions set at the beginning of the work of a minimalistic approach to using available resources and techniques in accordance with the ideas of green and sustainable chemistry.

The difference in the number of observed cells (initially, the number of displayed cells was the same in both cases) after 24 h comparing the two materials is significant (19.8 ± 0.8 times more cells per square cm based on the average of the cell counts for at least three frames and the statistically significant difference on the basis of a Mann–Whitney test *p* < 0.05), which indicates that the LMWChG10 material stimulates growth and division. This can be linked to differences in the contact angle of these materials, which, as reported in the literature, have a significant effect on cell adhesion and division [28]. There are no explicit differences in cell morphology for the two materials, and it matches the normal pattern for this cell line.

### 2.7. Preliminary Chemical Characterization of Obtained Materials Using IR Spectroscopy

In order to obtain a full picture of the characteristics of the new substrates proposed in this work, IR ATR for all obtained final materials and IR grazing angle for the LMWCh and LMWChG10 were performed. The purpose of this was to preliminary show whether the phenomena responsible for the differences in the properties of materials are the product of chemical reactions and whether these reactions occur throughout the whole volume of the sample or only on its surface. Therefore, ATR vs. grazing angle spectra were compared, where the first method surveys the entire sample, where volume and surface information can be obscured, and the second one provides a picture mainly of the surface. The spectra obtained for the ATR measurements do not differ from each other when it comes to the samples obtained in the presence of small additions of glycerin (10 and 20%) and without it (Figure S2), where well-defined polymer films have been obtained. The change in the spectral image only occurs for the spectra obtained for the highest addition of glycerin and material undergoing partial destabilization during drying. Very similar changes in the pattern of the IR spectrum were obtained by the grazing angle (Figure S3) technique. This can be explained by the fact that the observed chemical reaction occurs mostly at the surface for useful material variants (10 and 20%) and occurs most intensively in the case of a sample that undergoes destruction (50%). Analyzing the obtained IR grazing angle spectra (Figure S3) of the LMWCh and LMWChG10 samples, the strengthening of and presence of new C=O bands derived from the carbonyl groups was observed for the second material (among others, 1675 cm^−1^ and 1585 cm^−1^, derived from the amide group). Taking into account that the sample contains only chitosan and residues of acetic acid, glycerin, and water, the most likely reaction that could produce such an effect is a reaction of the NH_2_ groups of the polymer with the previously mentioned carboxylic acid. This is partially consistent with comparisons of IR spectra of chitosan and chitin in the literature [29]. In such a reaction, chitosan acetylation would be thermodynamically supported by the process of water removal from the sample (effectively removed along with glycerin during annealing) and would lead to the formation of chitin on its surface. Transformation of chitosan into chitin or chitosan with a lower degree of deacetylation would explain the effects associated with the changes in the material’s interaction with water, manifested by an increase in the contact angle (Table 1) and weakening of swelling (Figure 3). Transformation of the material into chitin or chitosan with a lesser degree of deacetylation also justifies the destructive phenomena to which the material containing the highest amount of glycerol is subjected during drying.

## 3. Conclusions

Each ingredient that is added to a biomaterial for use as a substrate for cell growth and that is ultimately implanted into the body as a fragment of an implant requires careful analysis for biocompatibility and biodegradability. Consideration should be given to its release from the material, further fate in the body, excretion pathways, formed metabolite interactions with substances naturally present in the body, and xenobiotics. All of this creates complex webs of dependencies whose untangling can hinder the admission of new materials to the market, not only for practical but also for formal and legal reasons. Therefore, it seems reasonable to operate on as few components as possible when designing new systems of this type, basing them on naturally occurring components in the environment, thus realizing the now common strategy of moving away from fossil fuel-based polymers.

The goal formulated in this work was to obtain crosslinker-free chitosan hydrogel precursors using only five reagents and simple procedures, which, after swelling, provide materials of satisfactory mechanical properties (better than those for materials containing AMPS, which is also described in this work) that promote colonization and growth of mammalian cells. The proposed innovative processing procedure involved the addition of glycerin, which, during annealing by evaporation, allowed us to obtain a material with significantly better properties than in a similar experiment without its addition. Particularly important here is the improvement in mechanical properties in the case of swollen materials. This results from chemical changes which, depending on the amount of glycerin, occur mostly on the surface (10, 20%) or for the whole volume/bulk of the material (50%). These changes, as we postulate, involve reversing the deacetylation of chitosan, which brings the material closer to the physicochemical properties of chitin. The reactions give, in the case of surface modification, a positive effect in the form of an increase in hydrophobicity (manifested by an increase in the contact angle and a decrease in swelling) and, consequently, promote improvements in the mechanical properties and stimulation of cell growth on the surface. However, in the case of large amounts of glycerin and significant modifications throughout the volume, we obtained a brittle material not suitable for molding.

## 4. Materials and Methods

### 4.1. Materials

Low-molecular-weight (50–190 kDa) and medium-molecular-weight (190–310 kDa) chitosan, 2-Acrylamido-2-methyl-1-propanesulfonic acid (AMPS) (99%), glycerin (98%), potassium carbonate (analytically pure), ethanol (96%), and 2,2′-azobis(2-methylpropionamidine) dihydrochloride (powder or granules, 97%) were purchased from Sigma/Aldrich (Darmstadt, Germany). The dialysis tube 3.5 kDa was purchased from Carl Roth (Karlsruhe, Germany).

### 4.2. Apparatus

The mechanical properties of the films, i.e., the tensile strength and Young’s modulus, were determined using a testing machine, Inspect Table Blue 5 kN (Hegewald&Peschke, Nossen, Germany). We also used the environmental scanning electron microscope, SEM Phenom World PRO (PIK INSTRUMENTS, Piaseczno, Poland). A FTIR Thermo Scientific Nicolet iS10 was adapted for ATR and grazing angle measurements (reflectance accessory set at 64). The AFM Dimension Icon microscope (Bruker, Billerica, MA, USA), operating in an air or fluid mode using the PeakForce Tapping QNM ^®^ mode, was also employed. An optical microscope eclipse LV100 (Nikon, Tokio, Japan), and a mechanical stirrer, Eurostar power control-visc P1 (IKA-Werke, Staufen im Breisgau, Germany), were also employed.

### 4.3. Materials Syntesis

#### 4.3.1. Unmodified Chitosan-Based Material (LMWCh and MMWCh)

A total of 4.00 g of chitosan (LMW or MMW) was added to 200 mL of a 1% acetic acid solution. The whole mixture was stirred with a mechanical stirrer (at maximum rpm) for 10 min. After this time, 80 mL of deionized water was added and stirred for a further 10 min until a homogeneous viscous solution was obtained. The resulting solution was dialyzed for 4 days against water, regularly replacing the deionized water and stirring continuously using a magnetic stirrer. After dialysis, the chitosan solution was transferred to a vessel serving as a mold (4 cm × 12 cm) and dried at 75 °C using a forced air-flow dryer. Then, the obtained polymer film was cooled to room temperature and immersed in a saturated solution of K_2_CO_3_ in ethanol for 5 min before being rinsed with ethanol to remove any residual salt. The obtained material was dried with a stream of warm air for about 5 min. Further processing of the chitosan films was carried out in order to further remove water and sterilize the material. The film was vacuum dried at 170 °C for 1 h and then left in a vacuum to cool to room temperature.

#### 4.3.2. Chitosan Materials Augmented with Glycerine During Processing (LMWChG)

The second type of material was obtained analogously to those in the subsection above (Section 4.3.1), with the difference being that appropriate amounts of glycerine were added (10, 20, and 50% of the dry mass of used chitosan, respectively, for LMWChG10, LMWChG20, and LMWCh50) to the chitosan solutions (the materials were made only for the LMW chitosan because it showed better mechanical properties and surface homogeneity) after dialysis, and the systems thus obtained were mixed thoroughly. Further processing was carried out in a similar manner to that in Section 4.3.1. The vacuum-drying system was additionally equipped with a condenser “cold finger” (a glass element immersed in liquid nitrogen). The liquid was collected during the drying of those materials and examined using ATR-IR to confirm that it mainly contained glycerin.

#### 4.3.3. 2-Acrylamido-2-Methylpropane Sulfonic Acid (AMPS) Containing Material (LMWChAMPS)

2-Acrylamido-2-Methylpropane Sulfonic Acid (AMPS), in the synthesis proposed below, has dual functions: it is used as a solvent (chitosan is insoluble in water without the addition of acid) and a crosslinking agent (radical polymerization/crosslinking monomer). The use of this additive should improve the properties of the polymer hydrogel while minimizing the number of reagents used during synthesis. A total of 4.00 g of LMW chitosan was mixed with 200 mL of 2% aqueous solution of AMPS using a mechanical stirrer (at maximum rpm) for 10 min. After 10 min, an additional 80 mL of water was added to the mixture, and mixing was continued until a viscous solution was obtained. The solution so obtained was transferred to a dialysis tube and dialyzed against water for 4 days. Then, 40 mg of 2,2′-azobis(2-methylpropionamidine) dihydrochloride dissolved in 1 mL of water was added to the solution. The resulting mixture was poured into a mold and dried in a forced air-flow dryer at 50 °C. Drying at this temperature was carried out until the thickness of the polymer film dropped to about 0.25 cm, at which point, the dryer’s temperature was raised to 75 °C to initiate polymerization. At this temperature, the material was dried until the polymer film detached itself from the mold.

### 4.4. Swelling and Contact Angle Measurements

Fragments of the films obtained from the finished materials and systems at the various stages of synthesis, measuring about 1 × 1 cm, were immersed in water for a specified period of time (5, 15, 25, and 35 min). After this time, the materials were initially dried to remove the unbound water by pressing them gently to filter paper and then weighed. After this measurement, the fragment was again immersed in a new portion of water until the next weight measurement. Decay of the material in the form of discontinuity was marked, and the experiment was terminated. The swelling coefficient of the tested materials was calculated based on the following formula:

$$s\left(t_{x}\right)=\frac{\Delta m}{m_{0}}*100\%=\frac{m_{x}-m_{0}}{m_{0}}*100\%$$

Δ*m*—the weight increase of the material after time t; *m*_0_—the initial mass of the material; *m_x_*—the mass of the material at time t; *S*(*t_x_*)—the swelling ratio at time t. The obtained results were collected in the form of plots as a function of time.

Contact angle measurements were performed using water for the dry and swollen samples (exposure to water for 35 min). The latter, before measurement, was dried on filter paper in the same way as for the weight measurements during the swelling coefficient experiment. Measurements were not made on films that were either not in the form of a flat film or that were degraded during swelling.

### 4.5. SEM and AFM Imaging of the Material Surfaces

The surfaces of the finished materials were imaged by SEM electron microscopy after being attached with conductive tape to an aluminum measuring table without further processing. AFM measurements in the air were performed using standard silicon nitride cantilevers (Scanasyst-Air, Bruker) with a nominal spring constant of 0.4 N/m and sharp tips with a nominal tip radius of 2 nm. Measurements in water were made using standard silicon nitride cantilevers (Scanasyst-Fluid+, Bruker) with a nominal spring constant of 0.7 N/m and sharp tips with a nominal tip radius of 2 nm. The topography and roughness (RMS) images were presented after first-order plane fitting to the 10 × 10 μm images at a scan resolution of 256 × 256. For adhesion and the DMT modulus, the values were given as the mean along with the standard deviation from the 10 × 10 μm images at a scan resolution of 256 × 256.

### 4.6. Experiments to Evaluate the Mechanical Durability of the Material

For this purpose, samples of 50 × 200 mm (width × length) were prepared, the pre-load force was 2 N, and the test speed was 100 mm/min. Here, we tested the as-prepared LMWCh, MMWCh, LMWChAMPS, and swollen LMWCh samples for 5 min in distilled water. The MMWCh samples, after swelling for 5 min in distilled water, were unsuitable for the proposed mechanical test here due to degradation during swelling. Here, we also investigated the initial tests of glycerine-modified LMWChG materials; for these pilot studies, the LMWChG10 and LMWChG20 samples were selected.

The mechanical parameters were calculated by averaging 10 measurements and were presented as the mean ± standard deviation.

### 4.7. Ability of the Material to Be Colonized by Mammalian Cells

Material fragments of 0.2 × 0.2 cm were, in a sterile manner, removed from the vacuum oven and transferred to serum-free medium (DMEM) for 1 h. Then, the medium was replaced with one that contained serum (10%), and the materials were left for 6 days (after 3 days, the medium was again replaced with a fresh one). B16-F10 (ATCC CRL-6475) cells were seeded onto the materials prepared in this way and, after 24 h, were visualized after staining with crystal violet. The number of cells was estimated after dissolving the dye bound to the cells in a dedicated solvent and by measuring the absorbance, which is proportional to the number of cells on the surface of the material. All procedures used for the cellular model studies are described in detail in our earlier paper [30].

## Figures and Tables

**Table 1:** Contact angle measurement results for studied materials.

	Dry Material [°]	Swollen Material [°]
LMWChAMPS	57 ± 12	------- ^1^
LMWCh	119 ± 21	5 ± 1
LMWChG10	73 ± 6	88 ± 8
LMWChG20	43 ± 1	78 ± 23
Modified polystyrene dedicated to cell culture	60 ± 7	59 ± 8

^1^ Immeasurable (droplet spreads over the surface completely).

**Table 2:** Values characterizing the mechanical properties of the tested materials: E—Young’s modulus, σM—tensile strength, εM—elongation at break, Fm—maximum force.

	E [MPa]	σM [MPa]	εM [%]	Fm [N]
LMWChAMPS	776.0 ± 80.4	13.6 ± 5.2	6.1 ± 5.9	77.1 ± 27.7
LMWCh	2870.9 ± 544.4	66.5 ± 18.0	2.7 ± 2.8	73.1 ± 19.8
MMWCh	3166.5 ± 471.7	53.83 ± 20.3	0.50 ± 0.51	52.5 ± 19.8
LMWChG10	1671.1 ± 392.8	45.75 ± 12.0	2.3 ± 2.6	59.5 ± 15.6
LMWChG20	1548.4 ± 124.9	46.64 ± 4.5	28.1 ± 5.9	50.0 ± 4.9
LMWCh (swollen)	8.4 ± 1.3	0.94 ± 0.40	1.1 ± 1.3	1.5 ± 0.6
LMWChG10 (swollen)	17.2 ± 5.6	0.92 ± 0.34	0.19 ± 0.18	1.5 ± 0.5

**Table 3:** Quantifiable parameters characterizing the surfaces of materials on the basis of AFM measurements.

	LMWCh	LMWChG10	LMWCh10 (Swollen)
Roughness (RMS) [nm]	4.94	25.4	16.5
Adhesion [nN]	1.4 ± 0.4	3.0 ± 1.2	8.9 ± 1.2
DMT modulus [MPa]	54,400 ± 20,023	275 ± 29	12.4 ± 6

## Data Availability

The original contributions presented in this study are included in the article/Supplementary Materials. Further inquiries can be directed to the corresponding author.

## Supplementary Materials

The following supporting information can be downloaded at: https://www.mdpi.com/article/10.3390/gels11070485/s1, Figure S1: AFM images and corresponding DMT modulus distributions for LMWCh (A), LMWChG10 (B), and LMWCh10 swollen (C). The top pictures are the AFM-scanned DMT modulus maps, and the down graphs are the corresponding probability distribution curves of the DMT moduli; Figure S2: IR ATR measurements of obtained LMW chitosan materials; Figure S3: IR grazing angle measurements of material surfaces. LMWCh red and LMWChG10 black graph.
