# Supplementary material for: Green Minimalistic Approach to Synthesize Chitosan-Based Durable Polymer Hydrogel Materials for Supporting Cell Growth
Source: Gels. 2025 Jun 23;11(7):485. doi: 10.3390/gels11070485 (PMC12294684; doi:10.3390/gels11070485)
Supplement: Supplementary file 1 [file gels-11-00485-s001.zip › gels-3697663-supplementary.pdf]

gels

MDPI

Type of the Paper Article

Green minimalistic approach to synthesize chitosan-based durable polymer hydrogel materials for supporting cell growth

Justyna Pawlik [1], Klaudia Borawska [2], Piotr Wieczorek [2,3], Kamil Kaminski[2,*]

[1] Faculty of Materials Science and Ceramics, Department of Glass Technology and Amorphous Coatings, AGH University of Science and Technology, 30 Mickiewicza Ave., 30-059 Krakow, Poland; pawlikj@agh.edu.pl
[2] Faculty of Chemistry, Jagiellonian University, Gronostajowa 2 St., 30-387 Krakow, Poland; piotr.wieczorek@doctoral.uj.edu.pl
[3] Doctoral School of Exact and Natural Sciences, Jagiellonian University, Lojasiewicza 11, 30-348 Krakow, Poland
piotr.wieczorek@doctoral.uj.edu.pl
* Correspondence: kaminski@chemia.uj.edu.pl;

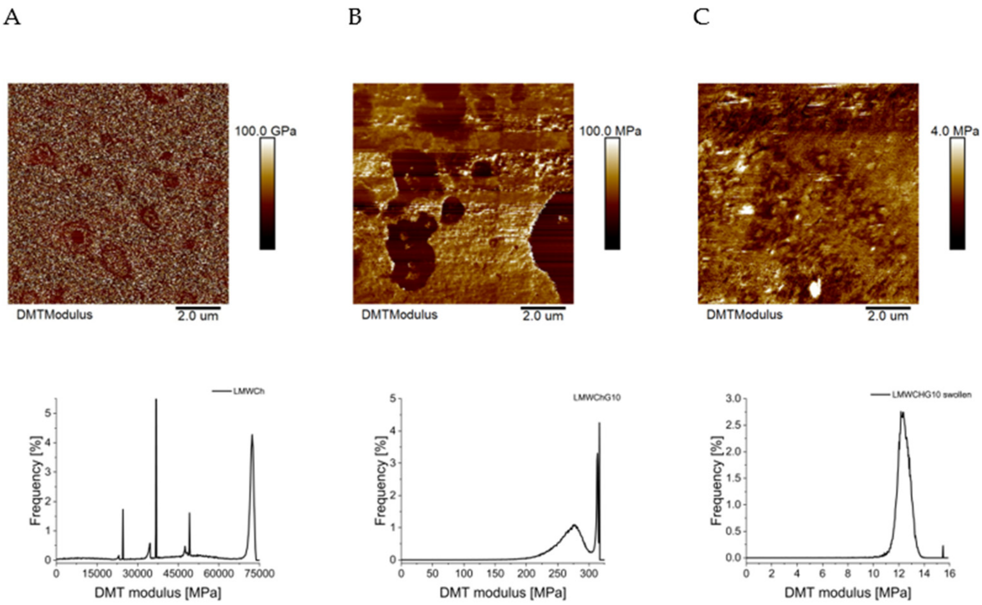

Figure S1. AFM images and corresponding DMT modulus distributions for LMWCh (A), LMWChG10 (B) and LMWCh10 swollen (C) . The top pictures are the AFM scanned DMT modulus maps, and the down graphs are the corresponding probability distribution curves of DMT modulus.

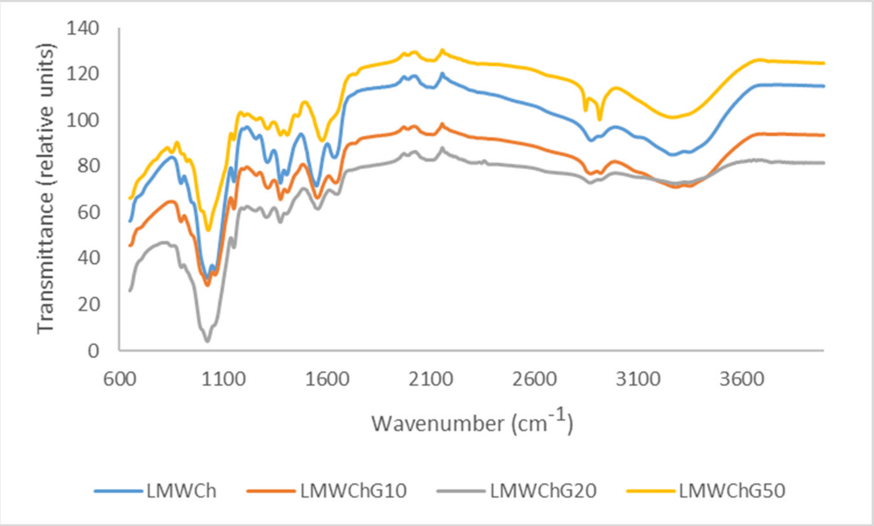

Figure S2. IR ATR measurements of obtained LMW chitosan materials.

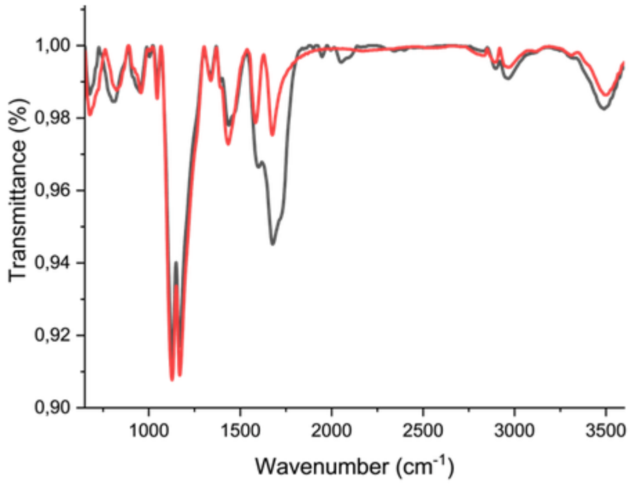

Figure S3. IR grazing angle measurements of material surfaces. LMWCh red and LMWChG10 black graph
